# Bayesian inversion of a diffusion model with application to biology

**DOI:** 10.1007/s00285-021-01621-2

**Published:** 2021-07-06

**Authors:** Jean-Charles Croix, Nicolas Durrande, Mauricio A. Alvarez

**Affiliations:** 1 School of Mathematical and Physical Sciences, University of Sussex, Falmer, Brighton BN1 9QH UK; 2 Prowler.io, Cambridge, CB2 1LA UK; 3 Department of Computer Science, University of Sheffield, Sheffield, S10 2TN UK

**Keywords:** Bayesian inverse problems, Diffusion equation, Functional MCMC, Gaussian processes, 62G05, 62P10, 35R30, 62F15

## Abstract

A common task in experimental sciences is to fit mathematical models to real-world measurements to improve understanding of natural phenomenon (reverse-engineering or inverse modelling). When complex dynamical systems are considered, such as partial differential equations, this task may become challenging or ill-posed. In this work, a linear parabolic equation is considered as a model for protein transcription from MRNA. The objective is to estimate jointly the differential operator coefficients, namely the rates of diffusion and self-regulation, as well as a functional source. The recent Bayesian methodology for infinite dimensional inverse problems is applied, providing a unique posterior distribution on the parameter space continuous in the data. This posterior is then summarized using a Maximum a Posteriori estimator. Finally, the theoretical solution is illustrated using a state-of-the-art MCMC algorithm adapted to this non-Gaussian setting.

## Introduction

The problem of diffusion in a porous media, which is ubiquitous in Physics, Engineering and Biology, is usually represented by the following partial differential equation (with constant diffusion and damping without transport):

1 $$\begin{aligned} \begin{aligned}&\frac{\partial z}{\partial t}(x,t)+\lambda z(x,t)-D\varDelta z(x,t)=f(x,t),\;\forall (x,t)\in \varOmega \times ]0,T],\\&z(x,t)=0,\;\forall (x,t)\in \varOmega \times \lbrace t=0\rbrace ,\\&z(x,t)=0,\;\forall (x,t)\in \partial \varOmega \times ]0,T],\\ \end{aligned} \end{aligned}$$

where the spatial domain is an open set $\varOmega \subset \mathbb {R}^n$ ($n\le 3$) and the final time is $T\in \mathbb {R}^+$ (other initial and boundary conditions are possible). In real world applications, the quantity of interest *z* (hereafter called the solution of Eq. 1) is typically the concentration of some chemical and evolves from a null initial state under three distinct mechanisms: (a) direct variation in concentration, given by the source *f*, (b) diffusion at rate *D*, (c) production or depletion at a rate $\lambda$. Different hypotheses on the parameters lead to a well-defined solution [well-posedness in the sense of Hadamard Hadamard (1902)] but only a particular case will be dealt with here. Besides the traditional computation of the solution from the parameters, one can use this model for the determination of an optimal control (e.g. source leading to the minimization of a particular cost functional) or the identification of parameters from partial knowledge of the solution in an inverse setting and the latter is the objective of this paper. The motivation comes from a challenging identification problem in Biology where the objective is to infer jointly self-regulation and diffusion rates with the source *f* given a limited number of noisy observations of the solution *z*. Note that given their physical interpretation, the parameters $u=(\lambda ,D,f)$ must all be non-negative (in an obvious sense).

This problem has already been solved using various approaches. In Becker et al. (2013), the authors use a system of ordinary differential equations instead of Eq. 1, and minimize a discrete version of a least-square penalty functional, while confidence intervals on parameters are given by bootstrapping. In a Bayesian setting, alternative methods have been based on Latent Force Models (Alvarez et al. 2013; Särkkä et al. 2018). These approaches assume that the source can be modelled with Gaussian Processes (Rasmussen and Williams 2006). In particular, if *f* is taken to be such a process and if the decay and the diffusion are constants, *z* is Gaussian as well (since *z* depends linearly in *f*). The two constant parameters $\lambda$ and *D* can then be optimized as hyper-parameters through standard likelihood maximization (Lopez-Lopera et al. 2019). Major drawbacks of this approach are both the difficulty to enforce positiveness of the reconstructed source and the absence of uncertainty quantification on $\lambda ,D$.

In this work, a more general methodology is applied, based on the recent advent of Bayesian Inverse Problems (Stuart 2010) for infinite dimensional spaces. This has the advantage of dealing with the ill-posedness while fully integrating the quantification of uncertainties in a unified approach. Moreover, the possibility to include previous physical constraints in the prior will be particularly meaningful. The rest of this paper is organized as follows: Sect. 2 presents all the mathematical analysis underlying the Bayesian inversion, namely sufficient regularity of the forward operator (mapping the parameter $(\lambda ,D,f)$ to the PDE solution *z*), existence and uniqueness of the posterior under a simple class of priors, and finally uniqueness of the associated posterior. Section 3 focuses on an adaptation of a geometric Markov Chain Monte-Carlo algorithm, well-defined in function spaces. Finally, Sect. 4 contains all implementation details, as well as a methodology to tune hyper-parameters of the model. All numerical results are based on a real-world dataset related to the developmental biology of the Drosophila Melanogaster.

## Bayesian inversion

As previously announced, the goal in this work is to infer a source term *f* (mRNA concentration) jointly with rates of diffusion *D* and decay $\lambda$ (i.e. the parameter $u=(\lambda ,D,f)$) from noisy and partial measurements of the solution *z* (gap protein concentration). This problem is ill-posed for multiple reasons: (a) the parameter *u* is infinite dimensional and only finite data are available, (b) the solution map is not injective and (c) observations are noisy. The typical approach to alleviate this issue is to regularize the problem, usually adding more constraints with Tikhonov-Philips functionals, to ensure uniqueness and continuity w.r.t. the observations (Isakov 2017; Schuster et al. 2012). Doing so, the regularized solution will be compatible with the dataset. Additionally, a particularly valuable information is a representation of all parameters *u* that would lead to similar data, giving precise statement on how the dataset is informative (Ghanem et al. 2017; Biegler et al. 2011; Sullivan 2015). One approach consists in treating these 2 objectives sequentially, first regularizing then quantifying the resulting uncertainty. However, the Bayesian methodology for inverse problems (Stuart 2010) and more recently Dashti and Stuart (2015) is precisely tailored to complete both tasks at once in an elegant manner. One particularity of these recent contributions is to tackle inverse problems directly in function spaces, postponing discretization at the very end for implementation purposes, which leads to robust algorithms w.r.t. discretization dimension. Indeed, finite approximations of probability measures may be absolutely continuous while their infinite counterparts are mutually singular. This becomes particularly troublesome in MCMC sampling for instance (Cotter et al. 2013).

In essence, instead of searching for one particular parameter solving the regularized problem, this approach considers conditional probability measures of parameters given observations. Namely, given a prior distribution (see Sect. 2.2) and few technical conditions on the forward operator (see Sect. 2.1), Bayes theorem applies and exhibits a unique posterior distribution (see Sect. 2.3), which is continuous in the data [w.r.t. Hellinger metric (Stuart 2010)]. Finally, one may summarize information from this posterior distribution with point estimators such as expected value or modes (Sect. 2.4). All these steps will now be presented.

### Forward model analysis

The first step is to detail precisely the required regularity of the solution map from Eq. 1. Using common variational techniques from PDE theory [see Evans (1998) or Brezis (2011)], one can show that this equation has a unique weak solution (see Proposition 1 which proof is in the appendix) given $u=(\lambda ,D,f)$ in a domain $\mathscr {U}$ that will be explicited later on. Moreover, this solution evolves smoothly when the parameter varies. Without any loss of generality and keeping in mind the biological application, the underlying physical domain will be $\varOmega =]0,L[$ with $L\in \mathbb {R}^+$, representing the anterior-posterior axis of a Drosophilia embryo.

#### Proposition 1

Let $0<\lambda _M$, $0<D_m\le D_M$, $\mathscr {U}=[0,\lambda _M]\times [D_m,D_M]\times \mathscr {C}([0,T]\times [0,L];\mathbb {R})$ with the norm $\Vert u\Vert _{\mathscr {U}}=\vert \lambda \vert +\vert D\vert +\Vert f\Vert _{\infty }$, then for all $u\in \mathscr {U}$, Eq. 1 has a unique weak solution *z*, continuous on the domain $[0,L]\times [0,T]$, defining the map:

$$\begin{aligned} z:u\in \mathscr {\mathscr {U}}\rightarrow z(u)\in C([0,T]\times [0,L];\mathbb {R}). \end{aligned}$$

Moreover, this map has the following properties:

1. (Energy estimate): it satisfies the following estimate $\forall u\in \mathscr {U}$,
  $$\begin{aligned} \Vert z(u)\Vert _{\infty }\le C\Vert f\Vert _{\infty }, \end{aligned}$$
  where $C>0$ is a constant independent of *u*,
2. (Local Lipschitz continuity): $\forall u$ in the interior of $\mathscr {U}$, $\forall r>0$ such that $\mathscr {B}(u,r)\subset \mathscr {U}$, $\exists L(u,r)>0$,
  $$\begin{aligned} \forall (u_1,u_2)\in \mathscr {B}(u,r)\times \mathscr {B}(u,r),\;\Vert z(u_1)-z(u_2)\Vert _{\infty }\le L(u,r)\Vert u_1-u_2\Vert _{\mathscr {U}} \end{aligned}$$
3. (Differentiability): it is twice Fréchet differentiable in the interior of $\mathscr {U}$.

The proof is standard using methods from PDE and optimal control theory (Evans 1998). The properties stated in proposition 1 will be important for subsequent analysis (Sect. 2.3):

1. The energy estimate is fundamental to establish most of the results concerning the posterior distribution. Indeed, it gives a precise upper bound, useful to show integrability of the yet-to-come likelihood,
2. Local Lipschitz continuity implies measurability of the solution map w.r.t. the Borel $\sigma$-algebra and is used in the characterization of posterior modes (Maximum a Posteriori estimators),
3. Second order Fréchet differentiability will be necessary for geometric methods in optimization (research of posterior modes) and sampling (Markov-Chain Monte-Carlo) since they rely on Hessian-type information.

### Choice of a prior distribution

The second step is to choose a prior probability distribution on $\mathscr {U}$, encoding all knowledge on the problem at hand, while being simple enough to keep the analysis tractable. The prior will be constructed as a product measure, specifying marginal measures $\mu _0^\lambda$, $\mu _0^D$, $\mu _0^f$ on all parameters:

2 $$\begin{aligned} \mu _0(du):=\mu _0^\lambda (d\lambda )\otimes \mu _0^D(dD)\otimes \mu _0^f(df). \end{aligned}$$

For simplicity, both measures for $\lambda$ and *D* will be taken uniform on respective intervals $[D_m,D_M]$ and $[0,\lambda _M]$. Now, since *f* must be non-negative, 1 is re-parametrized with the following source term:

3 $$\begin{aligned} f^*=\exp (f), \end{aligned}$$

where $f\in C([0,T]\times [0,L];\mathbb {R})$. Selecting a Borel probability measure $\mu _0^f$ on $C([0,T]\times [0,L];\mathbb {R})$ will imply both continuity and positivity of the source $f^*$ almost-surely. The energy estimate is adjusted:

$$\begin{aligned} \forall u\in \mathscr {U},\;\Vert z(u)\Vert _{\infty }\le C^*\exp \left( \Vert f\Vert _{\infty }\right) . \end{aligned}$$

In this work, $\mu ^f_0$ will be taken as the Gaussian measure associated with a continuous Gaussian process [see Bogachev (1998) for a presentation of infinite dimensional Gaussian measures] such that *f* is almost-surely continuous. Remark that the exponential map in Eq. 3 could be replaced with any sufficiently differentiable function from $\mathbb {R}$ to $\mathbb {R}^+$ (thus keeping the second order Fréchet differentiability of the solution map). Besides, alternative distributions are also possible for *f* like Besov priors [from Dashti et al. (2013)] or more general convex measures [from Hosseini and Nigam (2017)]. However, this choice is also motivated by practical reasons, since one can build a Gaussian measure $\mu _{ref}$ dominating $\mu _0$:

$$\begin{aligned} \mu _{ref}=\mathscr {N}(\lambda _{ref},\sigma _\lambda ^2)\otimes \mathscr {N}(D_{ref},\sigma _D^2)\otimes \mu _0^f. \end{aligned}$$

Indeed, choose $(\lambda _{ref},D_{ref})\in \mathbb {R}^2$ and $\sigma _\lambda ^2,\sigma _D^2>0$ then $\mu _0<<\mu _{ref}$ with

$$\begin{aligned} \begin{aligned}&\frac{d\mu _0}{d\mu _{ref}}(u)=\frac{d\mu _0^\lambda }{d\mu _{ref}^\lambda }(\lambda )\frac{d\mu _0^D}{d\mu _{ref}^D}(D)\\&\quad =\frac{2\pi \sigma _\lambda \sigma _D}{\lambda _M(D_M-D_m)}\exp \left( \frac{(\lambda -\lambda _{ref})^2}{2\sigma _\lambda ^2}+\frac{(D-D_{ref})^2}{2\sigma _D^2}\right) \mathbb {1}_{[0,\lambda _{ref}]}(\lambda )\mathbb {1}_{[D_m,D_M]}(D). \end{aligned} \end{aligned}$$

The parameters of $\mu _{ref}$ are tuned by choosing $\lambda _{ref}=\frac{\lambda _M}{2}$, $\sigma _\lambda ^2=\frac{\lambda _M^2}{12}$, $D_{ref}=\frac{D_M-D_m}{2}$ and $\sigma _D^2=\frac{(D_M-D_m)^2}{12}$ (minimizing Kullback-Leibler divergence of $\mu _{ref}$ relative to $\mu _0$). This dominant measure will be critical for posterior modes analysis (Sect. 2.4) and MCMC sampling (Sect. 3.2).

### Posterior distribution

It is now time to show that the particular setting provided so far (forward model and prior distribution) leads to a well defined posterior measure. This is the purpose of Proposition 2 which is a direct application of the theory initially developed in Stuart (2010) [see Dashti and Stuart (2015) for an updated presentation]. In this purpose, consider a dataset $y=(y_i)_{i\in [1,n]}\in \mathbb {R}^n$ consisting of observations from the solution *z* at different times and locations $(t_i,x_i)_{i\in [1,n]}\in \left( [0,T]\times [0,L]\right) ^n$, produced under the following additive noise model (in vector notations):

4 $$\begin{aligned} y=\mathscr {G}(u)+\eta , \end{aligned}$$

where $\eta \sim \mathscr {N}(0,\sigma _\eta ^2I_n)$ ($I_n$ being the identity matrix of dimension *n*) and $\mathscr {G}:\mathscr {U}\rightarrow \mathbb {R}^n$ is the observation operator, mapping directly the PDE parameter *u* to the value of the associated solution $(z[u](x_i,t_i))_{i\in [1,n]}$ (composition of solution map *z* with Dirac type measure). Note that it is well-defined since the function *z* is continuous on the domain for every $u\in \mathscr {U}$. The following proposition, which is again proved in the appendix, establishes the existence, uniqueness and continuity in *y* of the posterior probability measure $\mu ^y$ (the solution of the inverse problem), expressing how observations *y* updated prior beliefs $\mu _0$ on the parameter *u*.

#### Proposition 2

Let $\mathscr {G}$ be the observation operator defined in Eq. 4, $y\in \mathbb {R}^n$ a dataset and $\mu _0$ the probability measure defined in Eq. 2, then there exists a unique posterior measure $\mu ^y$ for $u\vert y$. It is characterized by the following Radon-Nikodym density w.r.t. $\mu _0$:

$$\begin{aligned} \forall u\in \mathscr {U},\;\frac{d \mu ^y}{d\mu _0}(u)=\frac{1}{Z(y)}\exp \left( -\varPhi (u;y)\right) , \end{aligned}$$

with the negative log-likelihood

$$\begin{aligned} \varPhi (u;y)=\frac{1}{2\sigma _\eta ^2}\Vert y-\mathscr {G}(u)\Vert _{\mathbb {R}^n}^2, \end{aligned}$$

and the marginal

$$\begin{aligned} Z(y)=\int _\mathscr {U}\exp (-\varPhi (u;y)\mu _0(du). \end{aligned}$$

Furthermore, $\mu ^y$ is continuous in *y* w.r.t. Hellinger distance.

In fact, Proposition 2 gives two distinct results: a) the existence and uniqueness of a posterior (as long as $\mu _0$ is Radon and $\mu _0(\mathscr {U})=1$, which is the case here), b) well-posedness of the Bayesian inverse problem. In particular, the use of a Gaussian prior on *f* gives sufficient integrability, even under the re-parametrization from Eq. 3 (using Fernique’s theorem). If one chooses a different map between $f^*$ and *f* in Eq. 3, this condition may be considerably relaxed (using something slower than the exponential) and prior measures with lower integrability conditions can be considered.

### Maximum a posteriori estimator

In the previous section, the well-posedness of the Bayesian inverse problem has been proved. However, the posterior distribution is only known up to a multiplicative constant, through its density w.r.t. $\mu _0$. In the application, $\mu ^y$ will need to be summarized, which is usually done by the selection of a particular estimator in $\mathscr {U}$, such the posterior mean or a mode. One consequence of Proposition 2 is that the posterior mean is automatically continuous in the data *y* [since well-posedness is w.r.t. Hellinger distance, see Dashti and Stuart (2015)]. However, optimality properties (in a decision theoretic context) are not yet well-understood in infinite dimension to the best of our knowledge. This is why posterior modes (or Maximum a Posteriori) are considered instead. Furthermore, they provide a clear link with the classical Tikhonov–Philips regularization [see Dashti et al. (2013); Helin and Burger (2015); Agapiou et al. (2018)] and a useful variational characterization (in case of Gaussian or Besov priors) which will be the cornerstone of the numerical application, see Proposition 3 (which proof is given in the appendix).

#### Proposition 3

Let $\mu _0$ be the prior probability measure defined in Eq. 2 and $\mu _{ref}$ the Gaussian reference measure from Eq. 2.2, then the modes of $\mu ^y$ are exactly the minimizers of the following (generalized) Onsager-Machlup functional:

$$\begin{aligned} I(u):=\varPhi (u;z)+\frac{1}{2}\Vert f\Vert ^2_{\mu _0^f}, \end{aligned}$$

where $\Vert .\Vert _{\mu _0^f}$ is the Cameron–Martin norm associated to $\mu _0^f$.

A minimizer of the previous generalized Onsager-Machlup functional will be noted $u_{MAP}=(\lambda _{MAP},D_{MAP},f_{MAP})$ and need not be unique. The precise application of this proposition to the biological setting is done in Sect. 4.1, once the prior distribution is fully specified.

### Approximation

The final step in the theoretical analysis of the Bayesian inverse problem is to study its approximation properties, since it will be solved numerically. There are two important things to check: (a) properties of the approximated posteriors (since it is what is available), (b) the consistency of these approximated posteriors. This will be done by projection of the parameter *u* onto a finite dimensional subspace of $\mathscr {U}$, constructed with a stochastic basis under $\mu _0^f$ [see Okazaki (1986) for a detailed introduction on stochastic bases including Banach spaces]. The second source of approximation is the use of a numerical solver for the PDE solution, but this will be neglected here (but can be conducted in a subsequent work). The next proposition will establish that the posterior distribution is well approximated, giving an estimate of the error when the Hilbert basis is chosen specifically (spectral basis of the covariance operator, namely Karhunen-Loève decomposition) and its proof is in the appendix.

#### Proposition 4

Let $\mu _0$ be the prior probability measure from 2, $\mathscr {G}$ the observation operator from Eq. 4, $y\in \mathbb {R}^n$ a dataset, $(f_n)_{n\in \mathbb {N}}\subset \mathscr {C}([0,L]\times [0,T];\mathbb {R})$ a stochastic basis for $\mu ^0_f$. Note $\forall m \in \mathbb {N}$, $P_mf$ the projection of *f* onto the span of $f_1,\dots ,f_m$ and $(\varPhi _m)_{m\in \mathbb {N}}$ the following sequence of approximate negative log-likelihoods:

$$\begin{aligned} \varPhi _m(u;y):=\varPhi ((\lambda ,D,P_mf);y), \end{aligned}$$

then the sequence $(\varPhi _m)_{m\in \mathbb {N}}$ defines a family of posterior measures $(\mu _m^y)_{m\in \mathbb {N}}$, all continuous w.r.t. *y* and such that

$$\begin{aligned} \lim _{m\rightarrow \infty }d_{Hell}(\mu ^y,\mu ^y_m)=0. \end{aligned}$$

## Metropolis–Hastings algorithm

As it was previously announced, the main motivation for the Bayesian methodology here is quantification of uncertainty, which will be done by sampling the posterior measure. Among the vast catalogue of methods for probability distributions simulation (Sequential Monte-Carlo, Approximate Bayesian Computations, Transport Maps, etc...), Markov chain Monte-Carlo is very popular [MCMC, see Brooks et al. (2011)] and well defined on function spaces Tierney (1998) even though ergodicity analysis of such algorithms is still in its infancy (Hairer et al. 2005, 2007, 2014; Rudolf and Sprungk 2018). After a short presentation of the Metropolis–Hastings algorithm (Sect. 3.1), this section will focus on a state-of-the-art Markov kernel designed to sample from Gaussian measures (Sect. 3.2) and adapt it to the current non-Gaussian prior using the Gaussian dominating measure $\mu _{ref}$.

### Metropolis–Hastings on function spaces

The Metropolis–Hastings algorithm (MH) is a very general (Tierney 1998) method to design Markov chains to sample from a given probability measure. It is based on a two-step process on each iteration:

1. Given a current state $u\in \mathscr {U}$, propose a new candidate *v* according to a proposal Markov kernel *Q*(*u*, *dv*) (it is a probability distribution on $\mathscr {U}$ for almost any $u\in \mathscr {U}$),
2. Accept the new state *v* with probability $\alpha (u,v)$ or remain at *u*.

This algorithm provides a sample distributed under a predefined probability measure $\mu$, if one selects $\alpha$ and *Q* in a specific way [see Tierney (1998) for a discussion in general state spaces]. For instance, let $\nu (du,dv)=\mu (du)Q(u,dv)$ and $\nu ^T(du,dv)=\nu (dv,du)$, the Metropolis–Hastings algorithm typically considers the following acceptance probability:

5 $$\begin{aligned} \alpha _{MH}(u,v)=\min \left( 1, \frac{d\nu ^T}{d\nu }(u,v)\right) , \end{aligned}$$

which, in particular, requires the absolute continuity of $\nu ^T$ w.r.t. $\nu$ (detailed balance condition of the Markov chain). Contrary to finite dimensional situations, this condition may be difficult to satisfy and a common way to overcome this situation in Bayesian Inverse problems [see Dashti and Stuart (2015); Girolami and Calderhead (2011); Beskos et al. (2017); Cotter et al. (2013); Hairer et al. (2014)] is to select *Q* revertible w.r.t. $\mu _0$. Indeed, in this case (with $\nu _0(du,dv)=\mu _0(du)Q(u,dv)$):

6 $$\begin{aligned} \frac{d\nu ^T}{d\nu }(u,v)=\frac{\frac{d\nu ^T}{d\nu ^T_0}(u,v)}{\frac{d\nu }{d\nu _0}(u,v)}=\frac{\frac{d\mu ^y}{d\mu _0}(v)}{\frac{d\mu ^y}{d\mu _0}(u)}=\exp \left( \varPhi (u;y)-\varPhi (v;y)\right) . \end{aligned}$$

In theory, the MH algorithm can be implemented with a large family of proposal kernels *Q*. In practice however, they need to be as efficient as possible and thus adapted to the problem at hand. Two common desirable properties for *Q* are:

- to adjust to locally mimic the target distribution $\mu ^y$,
- include a step size to tune acceptance probability to reasonable values.

These two properties may be used to trade-off self-correlation, acceptance rates and convergence speed to high interest areas of the parameter space. Next section presents a proposal *Q* with both properties to sample from a Gaussian prior distribution.

### Geometric MCMC under Gaussian reference

The specific Markov proposal kernel *Q*, tailored to sample distributions having a density w.r.t. a Gaussian measure $\mu _{ref}$ will now be presented. Most of the recent work on infinite dimensional MCMC methods is based on the following Langevin stochastic differential equation:

7 $$\begin{aligned} \frac{du}{dt}=-\frac{1}{2}K(u)\left( \mathscr {C}_{ref}^{-1}(u-u_{ref})+\nabla _u\varPhi (u;y)\right) +\sqrt{K(u)}\frac{dW}{dt}, \end{aligned}$$

where *K*(*u*) is a (possibly position-dependent) preconditioner, *W* a cylindrical Brownian motion and $\nabla _u\varPhi (u;y)$ the gradient in *u* of the negative log-likelihood. According to Beskos et al. (2017), a semi-implicit discretization of Eq. 7 leads to a Markov chain with the following kernel:

8 $$\begin{aligned} Q(u,dv)=\mathscr {N}\left( \rho (u-u_{ref})+u_{ref}+\sqrt{1-\rho ^2}\frac{\sqrt{h}}{2}g(u),\sqrt{1-\rho ^2}K(u)\right) , \end{aligned}$$

where $h>0$ is a step-size parameter, $\rho =\frac{1-h}{1+h}$ and:

$$\begin{aligned} g(u)=-K(u)\left[ (\mathscr {C}_{ref}^{-1}-K(u)^{-1})(u-u_{ref})+\nabla _u\varPhi (u;y)\right] . \end{aligned}$$

This dynamic explores the parameter space with a balance between Newton-type descent to zones of high density and Gaussian exploration. The philosophy behind this kernel is to use alternative Gaussian reference measures locally adapted to the posterior distribution, since it has been recently showed that higher efficiency is obtained from operator weighted proposals [Law (2014) and later generalized in Beskos et al. (2017) and Cui et al. (2016)]. Indeed, highly informative datasets may result in a posterior measure significantly different from the prior in likelihood-informed directions and non-geometric kernels (such as Independent sampler or preconditioned Crank-Nicholson for instance) become ineffective in this case. However, the infinite dimensional manifold Modified Adjusted Langevin Algorithm [$\infty$-mMALA from Beskos et al. (2017)] considers a specific preconditioner:

$$\begin{aligned} K(u)=\left( \mathscr {C}_{ref}^{-1}+H_\varPhi (u)\right) ^{-1}, \end{aligned}$$

where $H_\varPhi (u)$ is the Gauss–Newton Hessian operator of $\varPhi$, which locally adapts to the posterior. This kernel does not preserve the distribution $\mu ^y$ but is shown to be absolutely continuous w.r.t. the reference measure $\mu _{ref}$, almost-surely in *u* (under technical assumptions regarding *K*(*u*) linked with Feldman-Hajek theorem) and the Radon-Nikodym density is:

$$\begin{aligned} \frac{dQ(u,dv)}{d\mu _{ref}}(v)=\frac{dN\left( \frac{\sqrt{h}}{2}g(u),K(u)\right) }{dN(0,\mathscr {C})}\left( \frac{v-\rho (u-u_{ref})-u_{ref}}{\sqrt{1-\rho ^2}}\right) , \end{aligned}$$

and noting $w=\frac{v-\rho (u-u_{ref})-u_{ref}}{\sqrt{1-\rho ^2}}$ as it is done in Beskos et al. (2017), it finally comes:

$$\begin{aligned} \begin{aligned} \ln \left( \frac{dQ(u,dv)}{d\mu _{ref}}(u,v)\right)&=-\frac{h}{8}\vert K(u)^{-\frac{1}{2}}g(u)\vert ^2 +\frac{\sqrt{h}}{2}\langle K(u)^{-\frac{1}{2}}g(u),K(u)^{-\frac{1}{2}}w\rangle \\&\quad -\frac{1}{2}\langle w,H_\varPhi (u)w\rangle +ln\left( \left| \mathscr {C}_{ref}^{\frac{1}{2}}K(u)^{-\frac{1}{2}}\right| \right) . \end{aligned} \end{aligned}$$

Finally, the acceptance probability associated to the Markov kernel from Eq. 8 is

$$\begin{aligned} \alpha (u,v)=\min \left( 1,\frac{\frac{dQ}{d\mu _{ref}}(v,u)\frac{d\mu ^y}{d\mu _0}(v)}{\frac{dQ}{d\mu _{ref}}(u,v)\frac{d\mu ^y}{d\mu _0}(u)}\right) . \end{aligned}$$

This algorithm is well-defined on function spaces (reversibility is ensured w.r.t. $\mu _0$), thus it is robust to discretization as required. The $\infty$-mMALA proposal may be computationally expensive, as it requires to compute both gradient $\nabla \varPhi$, Gauss–Newton Hessian $H_\varPhi$ and the Cholesky decomposition of $K(u)^{-1}$ at each step. However, different dimension reduction techniques can be used [split in Beskos et al. (2017) or likelihood-informed in Cui et al. (2016)] to reduce the computational burden. A second alternative is to choose a constant preconditioner, located at a (precomputed) posterior mode for instance [similar to HMALA in Cui et al. (2016) and gpCN in Rudolf and Sprungk (2018)], which is done in this work.

## Numerical application

This section will now introduce the practical implementation of the previous methodology on the problem of reverse-engineering for post-transcriptional gap-gene in Drosophila Melanogaster. First, the prior distribution is detailed, as well as a random series representation for *f*, leading to an approximation of $f^*$. Moreover, the associated generalized Onsager–Machlup functional is also provided. Then, quantitative results are given on the dataset taken from Becker et al. (2013), consisting in protein concentration measurements irregularly spread in space and time.

### Choice of a continuous Gaussian process

In the previous analysis, $\mu _0^f$ has been defined as the probability measure over $\mathscr {C}([0,L]\times [0,T];\mathbb {R})$ associated with a continuous Gaussian process. In practice, it will be chosen centred for simplicity, thus completely specified by a covariance kernel over the space-time domain $[0,L]\times [0,T]$. The literature on such processes is vast [see Rasmussen and Williams (2006) for instance], and here a tensor product of two Brownian bridges (in time and space) will be used. The associated covariance kernel is given by:

9 $$\begin{aligned} K((x,t),(x',t'))=\sigma ^2\frac{4}{LT}\left( \min (x,x')-\frac{xx'}{L}\right) \left( \min (t,t')-\frac{tt'}{T}\right) , \end{aligned}$$

where the scaling constant is such that the process has maximum variance equal to $\sigma ^2$. In particular, this process has a well-known Karhunen–Loeve decomposition (see Bay and Croix) using Schauder-type hat functions. In turn, the process will thus be approximated as follows:

10 $$\begin{aligned} \begin{aligned}&\tilde{f}=\sum _{1\le i_1,i_2\le N}\sigma \sqrt{\lambda _{i_1,i_2}}\xi _i\varphi _{i_1,i_2},\\&\quad \varphi _{i_1,i_2}(x,t)=\varphi _{i_1}\left( \frac{x}{L}\right) \varphi _{i_2}\left( \frac{t}{T}\right) ,\;\forall (x,t)\in [0,L]\times [0,T],\\&\quad \lambda _{i_1,i_2}=\lambda _{i_1}\lambda _{i_2}, \end{aligned} \end{aligned}$$

where $(\xi _{i_1,i_2})_{1\le i_1,i_2\le N}$ are i.i.d. $\mathscr {N}(0,1)$ random variables and $\varphi _{i}$ are hat functions on dyadic intervals and the precise weights are given in Bay and Croix. We consider $N=20$ (400 basis functions) thus the approximated parameter $\tilde{u}=(\lambda ,D,\tilde{f})$ is of dimension 402.

### Solution map discretization

The analysis conducted in all previous sections happens to be valid for infinite dimensional quantities. In practice however, one needs to discretize for numerical experiments. In this work, the solution map is approximated using finite elements in space [FEniCS library in Python, see Alnaes et al. (2015) and Langtangen and Logg (2017)] and finite differences in time. We use 100 finite elements and 30 time steps on a 2018 standard laptop computer.^1^ We set $L=100$ and $T=100$ is the final time (data-points have been rescaled). All quantities related to negative log-likelihood derivatives (Gradient and Gauss–Newton Hessian matrix) are numerically computed using discrete adjoint methods [see Hinze et al. (2009) or Heinkenschloss (2008)] to keep scalability in *N*. The initial point in the chain is chosen at the MAP location, obtained by minimization of the functional in Eq. 3 (Prior based initialization results in long burnin phase). Practical optimization is done using L-BFGS-B algorithm from the Scipy library (Byrd et al. 1995).

### Estimation of noise variance

So far, the prior measure as well as the observation model include hyper-parameters playing fundamental roles and needing to be tuned. The complete list is $\lambda _M,D_m,D_M,\sigma _\eta ^2$ and to the best of our knowledge, there are no general methods to estimate them efficiently in this context. Indeed, no closed formulae exists for the likelihood (probability density of $\mathscr {G}(u)$) and cross-validation seems computationally out of reach. However, we provide here an empirical approach to tune the noise variance level $\sigma ^2_\eta$. The other parameters are fixed to arbitrary values. Our approach consists in:

1. Fix $$\hat{\sigma }_\eta ^2=1$$.
2. Find $u_{MAP}$ minimizing *I* using the current noise level $\sigma _\eta ^2$.
3. Update the current variance estimation $\hat{\sigma }_\eta ^2=\frac{1}{n-1}\Vert y-\mathscr {G}(u_{MAP})\Vert ^2$.
4. Go back to 2.

The algorithm is stopped once the parameter $\sigma _\eta ^2$ reaches a stable value, which in this application is $\hat{\sigma }_\eta ^2=20,50$. The estimator $u_{MAP}$ is taken as the last computed minimizer of the (discretized) Onsager-Machlup functional *I*.

### Results

**Fig. 1:**
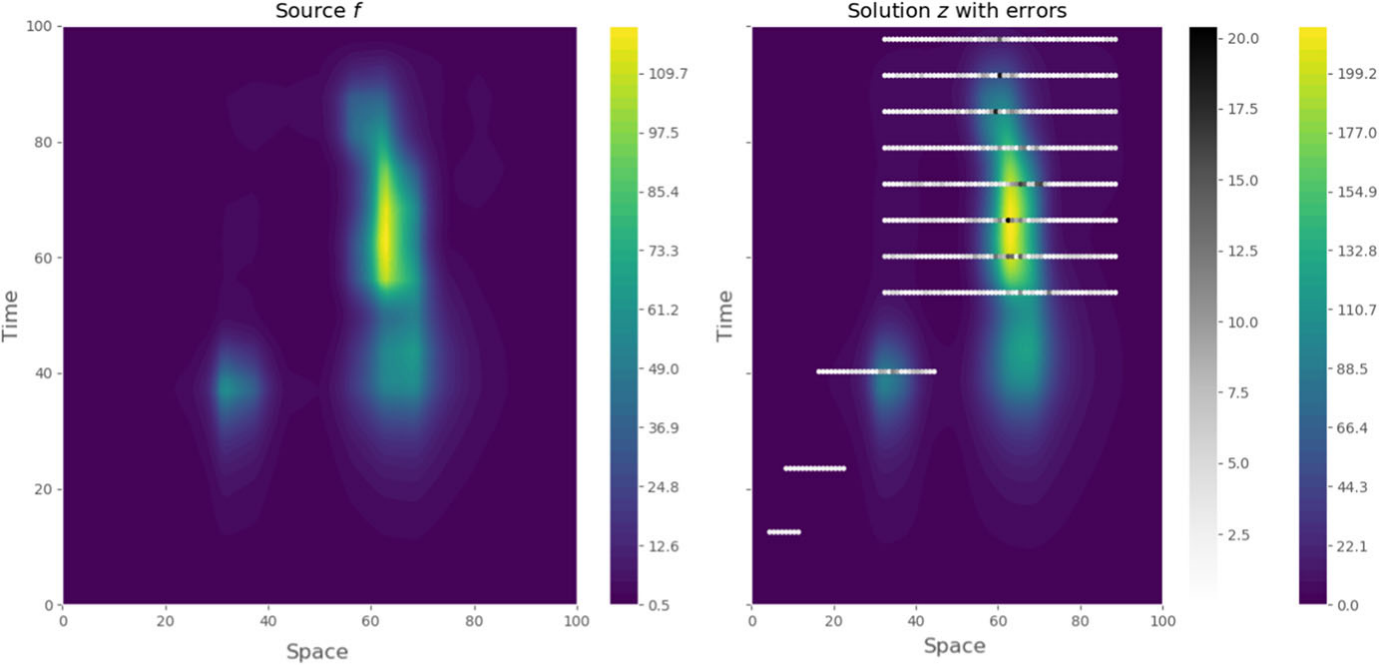
MAP estimator $u_{MAP}$ obtained from direct optimisation of the Onsager–Machlup functional. Left: estimated source term $f_{MAP}$ (using 400 basis functions). Right: estimated solution $z(u_{MAP})$ with absolute error at data locations. Grey bar represents the level of error between *y* and $\mathscr {G}(u_{MAP})$

We now turn to our main objective, the inversion and uncertainty quantification of gap-gene protein concentration from Becker et al. (2013). The dataset consists of 508 different measures which are non-uniformly spread in time and space (precise repartition can be seen in Fig. 1). With this estimated value, we compute our initial MAP estimate (numerical minimization of the Onsager-Machlup functional) and use it as initial point in the MCMC sampling. The Markov chain is ran for 21, 000 total iterations and the resulting traceplot is given in Fig. 4 for negative log-likelihood, decay, diffusion and first three components of *f*. The first thousand iterations are used as burnin and according to the autocorrelation function (Fig. 3), we choose to keep one iteration out of two hundreds as posterior sample (thinning). From this, we compute both posterior mean and MAP estimates, the precise values of decay, diffusion, negative log-likelihood and Onsager-Machlup functional being given in Table 1.

**Table 1:** Values of decay, diffusion, negative log-likelihood and Onsager–Machlup functional for MAP and posterior mean estimators

Parameter	$\lambda$	*D*	$\varPhi (u;z)$	*I*(*u*)
MAP (optimisation)	0.47	0.62	254.54	416.47
Posterior mean (sampling)	0.47	0.69	253.28	419.29

**Fig. 2:**
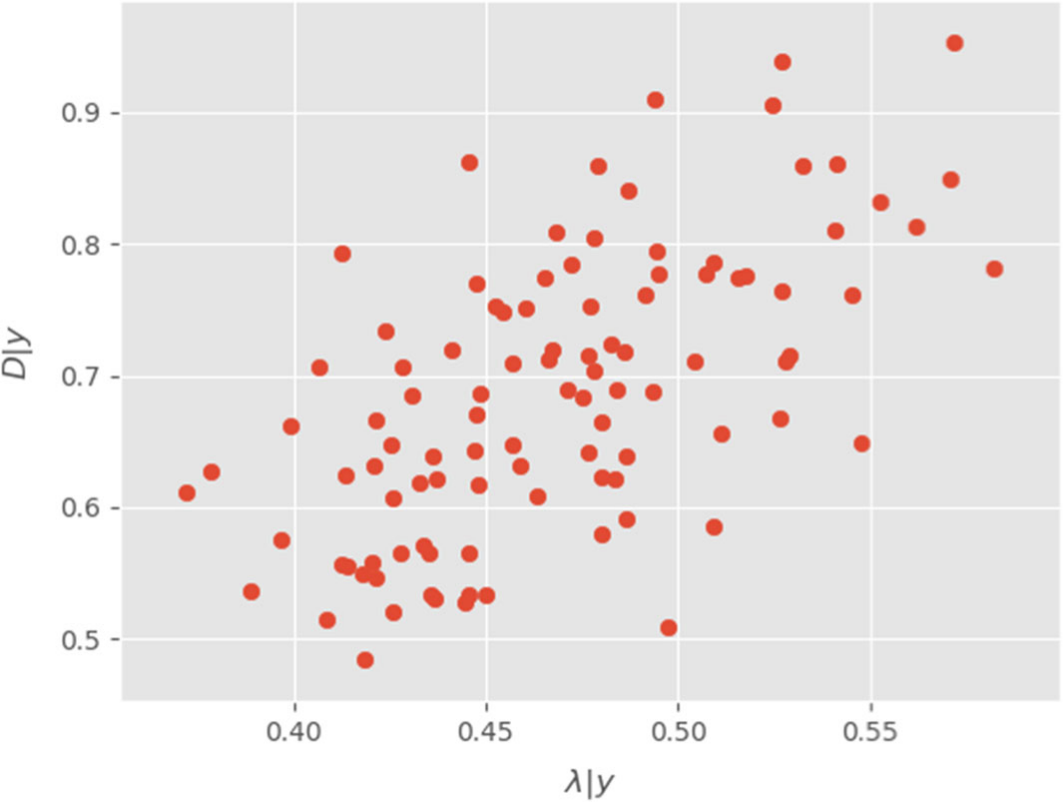
Samples from the marginal posterior distributions for $\lambda \vert y$ and $D\vert y$

Additionally to the estimated values, one can also look at the marginal distribution on Fig. 2. These values suggest that there is lack of identificability between $\varLambda$ and *D* (related to the non-injectivity of the forward operator). However, the strength of the Bayesian approach is to quantify this phenomenon, giving here potential values compatible with the data. Concerning the MAP estimator (Fig. 1), we recover both events described in Becker et al. (2013), that is 2 pikes of protein concentrations. The first happens on the anterior part of the embryo in the early experiment $(x=35,t=35)$. The second is much more intense and happens in the posterior part during the second half of the experiment. The estimated source explains these with an intense and localized increase in concentration. Finally, the uncertainty on both source and solution around data seems to be really low, which provides a good confidence on the level of mRNA at this time and part of the embryo (see Fig. 5). However, the point-wise variance on the solution *y* remains important before the first observations, which translates into an expected level of uncertainty. This indicates that given the data (and more precisely its time/space repartition), multiple scenarios are valid and our model would require more data to progress in these areas.

**Fig. 3:**
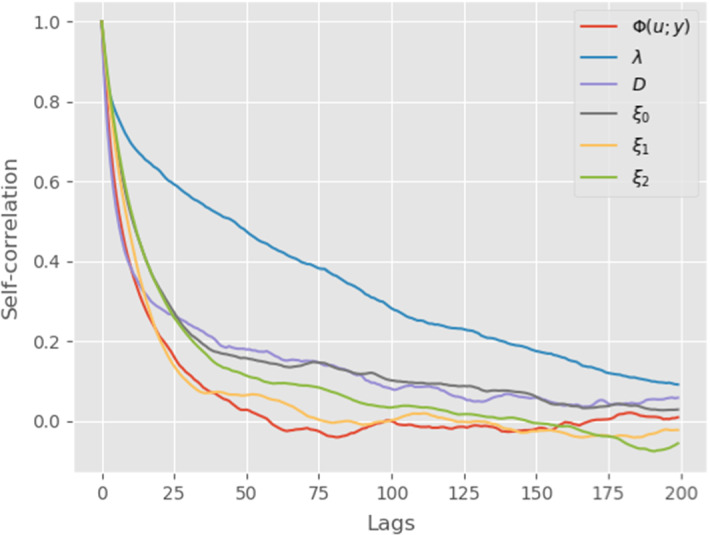
Self-correlation of $\varPhi (u;y)$, $\lambda$, *D*, $\xi _0$, $\xi _1$ and $\xi _2$, excluding the first 1000 iterations

**Fig. 4:**
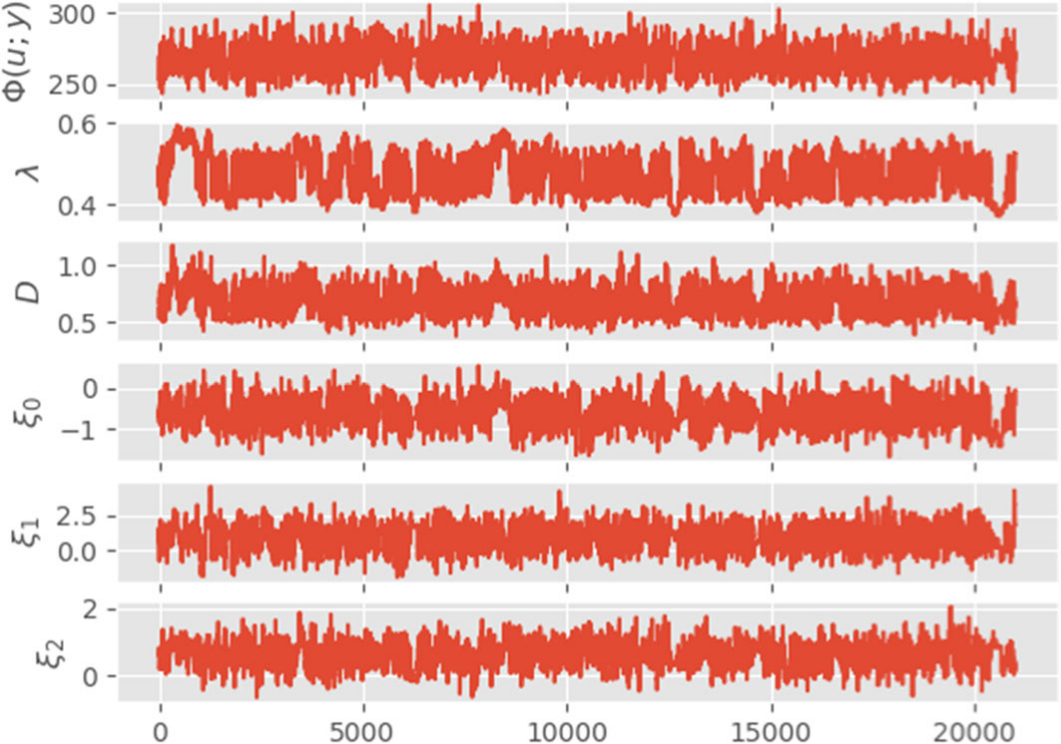
Trace plots of $\varPhi (u;y)$, $\lambda$, *D*, $\xi _0$, $\xi _1$ and $\xi _2$ (the first 1000 iterations are burned)

**Fig. 5:**
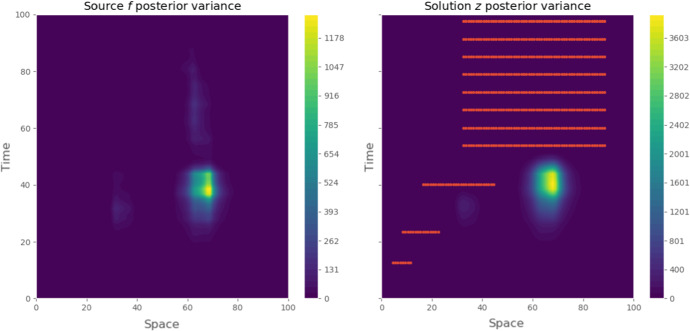
Posterior point-wise variance of $f^*\vert y$ (left) and $z(u)\vert y$ (right). Red dots indicate data locations

## Conclusions

In this work, we applied the Bayesian inverse problem methodology from Stuart (2010) to a practical biological dynamical system. Doing so, we provide a rigorous and detailed analysis of the forward model, existence and continuity of the posterior measure, characterization of MAP estimates, a consistent approximation and apply a state-of-the-art MCMC methodology. Because the forward MAP is non-linear, the uniqueness of posterior modes is unclear and it appears that local maximas are present. Nevertheless, the Bayesian methodology provides both a regularized solution to the problem, while giving a precious quantification of uncertainty. However, the estimation of prior hyper-parameters is still out of reach, giving poor confidence in the estimated variance. This direction requires further research, that we intend to address it in a future work.

## Proofs

### Proof of proposition 1

Using standard notations from PDE theory, let $\varOmega =]0,L[$, $H=L^2(\varOmega )$, $V=H^1_0(\varOmega )$, $V^*=H^{-1}(\varOmega )$ and

$$\begin{aligned} W=\left\{ z\in L^2([0,T],V),\;z'\in L^2([0,T],V^*)\right\} , \end{aligned}$$

equipped with the norm:

11 $$\begin{aligned} \Vert z\Vert _{W}=\left( \Vert z\Vert ^2_{L^2([0,T],V)}+\Vert z'\Vert ^2_{L^2([0,T],V^*)}\right) ^{\frac{1}{2}} \end{aligned}$$

1. (Existence, uniqueness and energy estimate) Let $\mathscr {U}=[0,\lambda _M]\times [D_m,D_M]\times \mathscr {C}([0,T]\times [0,L])$ with $\lambda _M>0$ and $0<D_m\le D_M<\infty$, then the differential operator in Eq. 1 is uniformly parabolic. By standard techniques, e.g. Galerkin approximation, one can show the existence and uniqueness of a weak solution $w\in W$ to Eq. 1 [Theorems 3 and 4, section 7.1, Evans (1998)]. Moreover, the regularity of the source term implies an improved regularity for the weak solution, namely $z\in L^2([0,T],H^2(\varOmega ))$ with $z'\in L^2([0,T],H)$ [Theorem 5, section 7.1, Evans (1998)]. The last result is an energy estimate:
  12 $$\begin{aligned} \Vert z\Vert _{L^2([0,T],H^2(\varOmega ))}+\Vert z'\Vert _{L^2([0,T],H)}\le C\Vert f\Vert _{L^2([0,T]\times [0,L])}. \end{aligned}$$
  Since *D* is bounded below by $D_m>0$, the constant *C* only on the coercivity property of the differential operator, thus $D_m$ and does not depend on *D*. This in turn implies the announced energy estimate, by the two continuous embeddings: $L^2([0,T]\times [0,L])$ in $\mathscr {C}([0,T]\times [0,L])$ and *z* into $\mathscr {C}([0,T]\times [0,L])$ [Theorem 4, section 5.9, Evans (1998)]:
  13 $$\begin{aligned} \Vert z\Vert _{\infty }\le C'\Vert f\Vert _{\infty }. \end{aligned}$$
2. (Local Lipschitz continuity in *u*, weaker norm) Let *u* in the interior of $\mathscr {U}$, $r>0$ such that $\mathscr {B}(u,r)\subset \mathscr {U}$ and $(u_1,u_2)\in \mathscr {B}(u,r)\times \mathscr {B}(u,r)$. There exists two unique solutions with respect to $u_1$ and $u_2$ such that $\forall i\in \lbrace 1,2\rbrace$, $\forall v\in L^2([0,T],V)$ and for almost-every *t* in [0, *T*]:
  $$\begin{aligned} \langle z_i'(t),v(t)\rangle _{V^*,V}+\lambda _i\langle z_i(t),v(t)\rangle _{H}+D_i\langle z_i(t),v(t)\rangle _{V}=\langle f_i(t),v(t)\rangle _{V^*,V}, \end{aligned}$$
  which, using subtraction and rearranging the terms, leads to:
  14 $$\begin{aligned} \begin{aligned}&\langle z_1'(t)-z_2'(t),v(t)\rangle _{V^*,V}+\lambda _1\langle z_1(t)-z_2(t),v(t)\rangle _{H}+D_1\langle z_1(t)-z_2(t),v(t)\rangle _{V}\\&\quad =\langle f_1(t)-f_2(t),v(t)\rangle _{V^*,V}+(\lambda _2-\lambda _1)\langle z_2(t),v(t)\rangle _{H}\\&\qquad +(D_2-D_1)\langle z_2(t),v(t)\rangle _{V}. \end{aligned} \end{aligned}$$
  Now, let $v=z_1-z_2$ in Eq. 14, drop the term $\lambda _1\Vert z_1-z_2\Vert _H^2$ in the left-hand side, use Cauchy–Schwarz and Poincaré’s inequalities in the right-hand side, then:
  $$\begin{aligned} \begin{aligned}&\langle z_1'(t)-z_2'(t),z_1(t)-z_2(t)\rangle _{V^*,V}+D_1\Vert z_1(t)-z_2(t)\Vert ^2_{V}\\&\quad \le \left( \Vert f_1-f_2\Vert _{V^*}+c\vert \lambda _2-\lambda _1\vert \Vert z_2\Vert _V+\vert D_2-D_1\vert \Vert z_2\Vert _{V}\right) \Vert z_1-z_2\Vert _V, \end{aligned} \end{aligned}$$
  where $c\ge 0$ is Poincaré’s constant. Integrating both sides between 0 and *T*, using the identity $\frac{d}{dt}\left( \frac{1}{2}\Vert z_1(t)-z_2(t)\Vert _H^2\right) =\langle z_1'(t)-z_2'(t),z_1(t)-z_2(t)\rangle _{V^*,V}$ and $z_1(0)=z_2(0)=0$ gives:
  15 $$\begin{aligned} \begin{aligned}&D_1\Vert z_1-z_2\Vert _{L^2([0,T],V)}^2\le \int _0^T\Vert f_1-f_2\Vert _{V^*}\Vert z_1-z_2\Vert _V dt\\&\quad +\left( c\vert \lambda _2-\lambda _1\vert +\vert D_2-D_1\vert \right) \int _{0}^{T}\Vert z_2\Vert _{V}\Vert z_1-z_2\Vert _Vdt. \end{aligned} \end{aligned}$$
  Now, it remains to use the identity $ab\le \frac{a^2}{2\epsilon ^2}+\frac{\epsilon ^2b^2}{2}$, that is
  $$\begin{aligned} \begin{aligned}&D_1\Vert z_1-z_2\Vert _{L^2([0,T],V)}^2\le \frac{1}{D_1}\Vert f_1-f_2\Vert _{L^2([0,T],V^*)}^2+\frac{D_1}{4}\Vert z_1-z_2\Vert _{L^2([0,T],V)}^2\\&\quad +\frac{\left( c\vert \lambda _2-\lambda _1\vert +\vert D_2-D_1\vert \right) ^2}{D_1}\Vert z_2\Vert _{L^2([0,T],V)}^2+\frac{D_1}{4}\Vert z_1-z_2\Vert _{L^2([0,T],V)}^2 \end{aligned} \end{aligned}$$
  which gives
  16 $$\begin{aligned} \Vert z_1-z_2\Vert _{L^2([0,T],V)}^2\le C(u,r)\Vert u_1-u_2\Vert _{\mathscr {U}}^2, \end{aligned}$$
  with $C(u,r)\ge 0$. Let’s rewrite equality 14 this way:
  17 $$\begin{aligned} \begin{aligned}&\langle z_1'(t)-z_2'(t),v(t)\rangle _{V^*,V}\\&\quad =-\lambda _1\langle z_1(t)-z_2(t),v(t)\rangle _{H}-D_1\langle z_1(t)-z_2(t),v(t)\rangle _{V}\\&\qquad +\langle f_1(t)-f_2(t),v(t)\rangle _{V^*,V}+(\lambda _2-\lambda _1)\langle z_2(t),v(t)\rangle _{H}\\&\qquad +(D_2-D_1)\langle z_2(t),v(t)\rangle _{V}. \end{aligned} \end{aligned}$$
  Applying Cauchy–Schwarz and Poincaré’s inequalities in the right-hand side of Eq. 17 gives:
  18 $$\begin{aligned} \begin{aligned}&\langle z_1'(t)-z_2'(t),v(t)\rangle _{V^*,V}\\&\quad \le \left( \left( \lambda _1+D_1\right) \Vert z_1(t)-z_2(t)\Vert _V+\Vert f_1(t)-f_2(t)\Vert _{V^*}+c\vert \lambda _2\right. \\&\qquad \left. -\lambda _1\vert \Vert z_2(t)\Vert _V\right) \Vert v(t)\Vert _{V}+\vert D_2-D_1\vert \Vert z_2(t)\Vert _V\Vert v(t)\Vert _{V}. \end{aligned} \end{aligned}$$
  It remains to integrate, take the supremum for *v* in the unit ball of $L^2([0,T],V)$ and use the previous estimate from Eq. 16 to conclude that
  $$\begin{aligned} \Vert z_1'-z_2'\Vert _{L^2([0,T],V^*)}^2\le C'(u,r)\Vert u_1-u_2\Vert _{\mathscr {U}}^2 \end{aligned}$$
  where $$C'(u,r)\ge 0$$.
3. (Local Lipschitz continuity in *u*, stronger norm). The previous analysis will be used to provide a similar result under a stronger norm. Indeed, the solutions are both satisfying $z_i\in L^2([0,T],H^2(\varOmega ))$ with $z_i'\in L^2([0,T], V)$, $\forall i\in \lbrace 1,2\rbrace$. Consider again Eq. 16, then it is equivalent to:
  19 $$\begin{aligned} \begin{aligned}&\lambda _1\langle z_1(t)-z_2(t),v(t)\rangle _{H}+D_1\langle z_1(t)-z_2(t),v(t)\rangle _{V}\\&\quad =\langle f_1(t)-f_2(t)+z_2'(t)-z_1'(t)+(\lambda _2-\lambda _1)z_2(t)\\&\qquad -(D_2-D_1)(z_2)_{xx}(t),v(t)\rangle _{H}. \end{aligned} \end{aligned}$$
  Using theorem 5 p. 323 in Evans (1998), one has:
  20 $$\begin{aligned} \begin{aligned}&\Vert z_1(t)-z_2(t)\Vert _{H^2(\varOmega )}^2\\&\quad \le C\left( \Vert z_1(t)-z_2(t)\Vert _{L^2(\varOmega )}^2+\Vert f_1(t)-f_2(t)+z_2'(t)-z_1'(t)\right. \\&\quad \left. +(\lambda _2-\lambda _1)z_2(t)-(D_2-D_1)(z_2)_{xx}(t)\Vert _{L^2(\varOmega )}\right) , \end{aligned} \end{aligned}$$
  which in turn, using integration, triangle inequality and the estimates obtained before leads to
  21 $$\begin{aligned} \Vert z_1-z_2\Vert _{L^2([0,T],H^2(\varOmega ))}\le C''(u,r)\Vert u_1-u_2\Vert _\mathscr {U}, \end{aligned}$$
  with $C''(u,r)\ge 0$. Consider one last time the weak formulation from Eq. 14, equivalent to (using integration by parts):
  22 $$\begin{aligned} \begin{aligned}&\langle z_1'(t)-z_2'(t),v(t)\rangle _{H}+\lambda _1\langle z_1(t)-z_2(t),v(t)\rangle _{H}-D_1\langle z_1(t)-z_2(t),v(t)\rangle _{H}\\&\quad =\langle f_1(t)-f_2(t),v(t)\rangle _{H}+(\lambda _2-\lambda _1)\langle z_2(t),v(t)\rangle _{H}\\&\qquad -(D_2-D_1)\langle z_2(t),v(t)\rangle _{H}. \end{aligned} \end{aligned}$$
  Now, choosing $v(t)=z_1'(t)-z_2'(t)$ gives:
  23 $$\begin{aligned} \begin{aligned}&\Vert z_1'(t)-z_2'(t)\Vert _{H}^2+\lambda _1\frac{d}{dt}\left( \frac{1}{2}\Vert z_1(t)-z_2(t)\Vert _{H}^2\right) \\&\quad =\langle f_1(t)-f_2(t),z_1'(t)-z_2'(t)\rangle _{H}+(\lambda _2-\lambda _1)\langle z_2(t),z_1'(t)-z_2'(t)\rangle _{H}\\&\qquad -(D_2-D_1)\langle (z_2)_{xx}(t),z_1'(t)-z_2'(t)\rangle _{H}+D_1\langle (z_1)_{xx}(t)\\&\qquad -(z_2)_{xx}(t),z_1'(t)-z_2'(t)\rangle _{H}. \end{aligned} \end{aligned}$$
  Using integration and Cauchy–Schwarz inequality, one obtains
  24 $$\begin{aligned} \Vert z_1'(t)-z_2'(t)\Vert _{L^2([0,T],H)}\le C'''(u,r)\Vert u_1-u_2\Vert _\mathscr {U}, \end{aligned}$$
  which concludes the proof on the local Lipschitz continuity. Finally, the continuous embedding of $\mathscr {C}([0,L]\times [0,T],\mathbb {R})$ into the more regular space, gives the result.
4. (Fréchet differentiability) Now, we deal with the Fréchet differentiability. Let $u=(\lambda ,D,f)$ in the interior of $\mathscr {U}$, $h^u=(h^\lambda ,h^D,h^f)\in \mathscr {U}$ such that $u+h^u\in \mathscr {U}$ and $h^z\in W$, then $\forall v\in L^2([0,T],V)$, we will use the following notation:
  25 $$\begin{aligned} \begin{aligned}&\left\langle F(z,u),v\right\rangle \\&\quad =\int _{0}^{T}\langle z'(t),v(t)\rangle _{V^*,V}dt+\lambda \int _{0}^{T}\langle z(t),v(t)\rangle _{V}dt\\&\quad +D\int _{0}^{T}\langle z(t),v(t)\rangle _{H}dt-\int _{0}^{T}\langle f(t),v(t)\rangle _{V^*,V}dt, \end{aligned} \end{aligned}$$
  then
  $$\begin{aligned} \langle F(z+h^z,u+h^u)-F(z,u),v\rangle =\langle F'(z,u)[h^z,h^u],v\rangle +c(h^u,h^z), \end{aligned}$$
  with (using triangle, Poincaré’s and Cauchy-Schwarz inequalities):
  $$\begin{aligned} \begin{aligned} \vert c(h^u,h^z)\vert&=\left| h^\lambda \int _{0}^{T}\langle h^z(t),v(t)\rangle _H dt+h^D\int _0^T\langle h^z(t),v(t)\rangle _V dt\right| \\&\le C\Vert v\Vert _{L^2([0,T],V)}\Vert h^u\Vert _\mathscr {U}\Vert h^z\Vert _{L^2([0,T],V)}\\&\le C\Vert v\Vert _{L^2([0,T],V)}\Vert h^u\Vert _\mathscr {U}\Vert h^z\Vert _{W} \end{aligned} \end{aligned}$$
  where $C\ge 0$ is a (new) constant independent from *u* and:
  $$\begin{aligned} \begin{aligned}&\langle F'(z,u)[h^z,h^u],v\rangle \\&\quad =\int _{0}^{T}\langle (h^z)'(t),v(t)\rangle _{V^*,V}dt+h^\lambda \int _{0}^{T}\langle z(t),v(t)\rangle _Hdt+h^D\int _{0}^{T}\langle z(t),v(t)\rangle _Vdt\\&\quad +\lambda \int _{0}^{T}\langle h^z(t),v(t)\rangle _Hdt+D\int _{0}^{T}\langle h^z(t),v(t)\rangle _Vdt-\int _{0}^{T}\langle h^f(t),v(t)\rangle _{V^*,V}dt. \end{aligned} \end{aligned}$$
  Using again Cauchy–Schwarz and Poincaré’s inequalities, we have:
  $$\begin{aligned} \vert \langle F'(z,u)[h^z,h^u],v\rangle \vert \le C\Vert (h^u,h^z)\Vert \Vert v\Vert _{L^2([0,T],V)} \end{aligned}$$
  with $C\ge 0$ another constant independent of $(h^z,h^u)$ thus $F'(z,u)$ is linear and bounded, which means that *F* is Fréchet-differentiable on $W\times \mathscr {U}$. Consider $F_z$ the partial derivative of *F* w.r.t. its first variable and $h^z\in W$, then $\forall v\in L^2([0,T],V)$:
  $$\begin{aligned} \begin{aligned} \langle F_z(z,u)h^z,v\rangle&=\int _{0}^{T}\langle (h^z)'(t),v(t)\rangle _{V^*,V}dt\\&\quad +\lambda \int _{0}^{T}\langle h^z(t),v(t)\rangle _Hdt+D\int _{0}^{T}\langle h^z(t),v(t)\rangle _V dt. \end{aligned} \end{aligned}$$
  This is the weak form associated to the same evolution problem (since *F* is linear in *z*), which thus has a unique weak solution $h^z\in W$, thus $F_z^{-1}$ exists. The previous energy estimate gives that $F_z^{-1}$ is bounded. Because *F* is differentiable and $F_z^{-1}$ exists and is bounded, the implicit function theorem applies and *z* is differentiable on $\mathscr {U}$ for the norm of *W*. The second order differentiability is obtained similarly so is the final embedding into the supremum norm.

$$\square $$

### Proof of proposition 2

In this proof, the conclusion will follow both theorems 4.3 and 4.5 from Dashti and Stuart (2015), which require to show that the following assumption is satisfied: $\varPhi (u;y)\in \mathscr {C}(\mathscr {U}\times \mathbb {R}^n;\mathbb {R})$, there exists two positive functions $M_i:\mathbb {R}^+\times \mathbb {R}^+$, monotonic non-decreasing in each variable, with $M_2>0$, such that $\forall r>0$, $\forall u\in \mathscr {U}$, $\forall y,y_1,y_2\in \mathscr {B}(0,r)$:

1. $$\varPhi (u;y)\ge -M_1(r,\Vert u\Vert _{\mathscr {U}})$$,
2. $$\vert \varPhi (u;y_1)-\varPhi (u;y_2)\vert \le M_2(r,\Vert u\Vert _{\mathscr {U}})\Vert y_1-y_2\Vert _{\mathbb {R}^n}$$.

Here, the the negative log-likelihood is:

26 $$\begin{aligned} \varPhi (u;y)=\frac{1}{2\sigma _\eta ^2}\Vert y-\mathscr {G}(u)\Vert _{\mathbb {R}^n}^2, \end{aligned}$$

thus it is clear that $\varPhi \in \mathscr {C}(\mathscr {U}\times \mathbb {R}^n;\mathbb {R}^+)$ (since $\mathscr {G}$ is continuous) and furthermore $M_1=0$ is a valid choice. To derive a valid function $M_2$, first observe that:

$$\begin{aligned} \forall u\in \mathscr {U},\;\Vert \mathscr {G}(u)\Vert _{\mathbb {R}^n} \le n\Vert z(u)\Vert _{\infty } \le C\exp \left( \Vert u\Vert _{\mathscr {U}}\right) . \end{aligned}$$

Consequently, $$\forall r>0$$ and $$\forall y_1,y_2\in \mathscr {B}(0,r)\subset \mathbb {R}^n$$, $$\forall u\in \mathscr {U}$$:

$$\begin{aligned} \begin{aligned} \vert \varPhi (u;y_1)-\varPhi (u;y_2)\vert&=\frac{1}{2\sigma _\epsilon ^2}\left| \Vert y_1\Vert _{\mathbb {R}^n}^2-\Vert y_2\Vert _{\mathbb {R}^n}^2+2\langle y_2-y_1,\mathscr {G}(u)\rangle _{\mathbb {R}^n}\right| \\&=\frac{1}{2\sigma _\epsilon ^2}\left| (\Vert y_1\Vert _{\mathbb {R}^n}-\Vert y_2\Vert _{\mathbb {R}^n})(\Vert y_1\Vert _{\mathbb {R}^n}+\Vert y_2\Vert _{\mathbb {R}^n})\right. \\&\quad \left. +2\langle y_2-y_1,\mathscr {G}(u)\rangle _{\mathbb {R}^n}\right| \\&\le \frac{1}{\sigma _\eta ^2}(r+\Vert \mathscr {G}(u)\Vert _{\mathbb {R}^n})\Vert y_1-y_2\Vert _{\mathbb {R}^n},\\&\le \frac{1}{\sigma _\eta ^2}\left( r+C\exp \left( \Vert u\Vert _\mathscr {U}\right) \right) \Vert y_1-y_2\Vert _{\mathbb {R}^n},\\&\le M_2(r,\Vert u\Vert _\mathscr {U})\Vert y_1-y_2\Vert _{\mathbb {R}^n}. \end{aligned} \end{aligned}$$

It remains to show that:

27 $$\begin{aligned} (1+M_2(r,\Vert u\Vert _\mathscr {U})^2)\in L^1(\mathscr {U},\mathscr {B}(\mathscr {U}),\mu _0), \end{aligned}$$

which is a direct consequence of Fernique’s and Fubini’s theorems, since $\mu _0^f$ is Gaussian. Finally, all conditions from Dashti and Stuart (2015) are verified, leading to existence, uniqueness and continuity of the posterior distribution. $\square$

### Proof of proposition 3

In this work, the prior measure $\mu _0$ is non-Gaussian. However, it might be seen as a small departure from a Gaussian measure of reference, $\mu _{ref}$ and this provides a useful variational characterization of modes. Indeed, one has

28 $$\begin{aligned} \frac{d\mu ^y}{d\mu _{ref}}(u)=\frac{d\mu ^y}{d\mu _0}(u)\frac{d\mu _0}{d \mu _{ref}}(u)\propto \exp \left( -\frac{1}{2\sigma _\eta ^2}\Vert y-\mathscr {G}(u)\Vert ^2-\frac{1}{2}\Vert f\Vert _{\mu _0^f}^2\right) \mathbb {1}_{[0,\lambda _{ref}]}(\lambda )\mathbb {1}_{[D_m,D_M]}(D).\nonumber \\ \end{aligned}$$

Now, since $\varPhi$ is locally Lipschitz continuous in *u* (as a consequence of proposition 1), the analysis from section 4.3 in Dashti and Stuart (2015), initially valid under Gaussian priors, apply as is. $\square$

### Proof of proposition 4

First of all, let us show that the sequence of maps $(\varPhi _m)_{m\in \mathbb {N}}$ provides a well-defined sequence of posterior measures $(\mu ^y_m)$, all continuous in the data w.r.t. Hellinger metric. The fundamental tool here, is the existence of a Banach space $E\subset \mathscr {C}([0,L]\times [0,T])$, continuously embedded, where $(f_m)_m$ is a Schauder basis and such that $\mu _0^f(E)=1$ [Theorem 2 in Okazaki (1986)]. This implies that $\mu _0$-almost surely,

29 $$\begin{aligned} \forall m\in \mathbb {N},\;\Vert P_mf\Vert _\infty \le C\Vert P_mf\Vert _E\le C'\Vert f\Vert _E. \end{aligned}$$

In particular, since Fernique’s theorem applies for the norm of *E*, the analysis from Proposition 2 applies to all measures $\mu _m$. Now, to see that the approximation is consistent, we will apply theorem 4.8 in Dashti and Stuart (2015), which requires to show that

30 $$\begin{aligned} \vert \varPhi (u;y)-\varPhi _m(u;y)\vert \le M(\Vert u\Vert _\mathscr {U})\psi (m) \end{aligned}$$

where $\lim _{m\rightarrow \infty }\psi (m)=0$ and $1+M^2(\Vert u\Vert _\mathscr {U})\in L^1(\mu _0)$. Now, noting $u_m=(\lambda ,D,P_mf)$ it comes:

31 $$\begin{aligned} \Vert \mathscr {G}(u_m)\Vert _{\mathbb {R}^n}\le n\Vert z(u_m)\Vert _{\infty }\le C\exp \left( \Vert P_mf\Vert _\infty \right) \le C_1\exp \left( C_2\Vert f\Vert _E\right) . \end{aligned}$$

This now gives $\forall y\in \mathscr {B}(0,r)$:

$$\begin{aligned} \begin{aligned}&\vert \varPhi (u;y)-\varPhi _m(u;y)\vert \\&\quad =\frac{1}{2\sigma _\eta ^2}\left| \Vert G(u)\Vert _{\mathbb {R}^n}^2-\Vert \mathscr {G}(u_m) \Vert _{\mathbb {R}^n}^2+2\langle y,\mathscr {G}(u_m)-\mathscr {G}(u)\rangle _{\mathbb {R}^n}\right| \\&\quad =\frac{1}{2\sigma _\eta ^2}\left| (\Vert \mathscr {G}(u)\Vert _{\mathbb {R}^n}-\Vert \mathscr {G}(u_m)\Vert _{\mathbb {R}^n})(\Vert \mathscr {G}(u)\Vert _{\mathbb {R}^n}+\Vert \mathscr {G}(u_m)\Vert _{\mathbb {R}^n})\right. \\&\qquad \left. +2\langle y,\mathscr {G}(u_m)-\mathscr {G}(u)\rangle _{\mathbb {R}^n}\right| \\&\quad \le \frac{1}{\sigma _\eta ^2}(r+C_1\exp \left( C_2\Vert f\Vert _E\right) \left\Vert \mathscr {G}(u)-\mathscr {G}(u_m)\right\Vert _{\mathbb {R}^n}. \end{aligned} \end{aligned}$$

Now, one can use the linearity of *z* in $f^*$, thus

32 $$\begin{aligned} \left\Vert \mathscr {G}(u)-\mathscr {G}(u_m)\right\Vert _{\mathbb {R}^n}\le n\Vert z(u)-z(u_m)\Vert _\infty \le C\Vert \exp (f)-\exp (P_mf)\Vert _\mathscr {\infty }. \end{aligned}$$

It remains to see that

33 $$\begin{aligned} \Vert \exp (f)-\exp (P_mf)\Vert _\mathscr {\infty }\le C\exp (\Vert f\Vert _E)\Vert f-P_mf\Vert _E \end{aligned}$$

and since $\Vert f-P_mf\Vert _E\rightarrow 0$, the result is clear as Fernique’s theorem in *E* gives the required integrability. $\square$

## Footnotes

1 All codes are available online at https://github.com/JeanCharlesCroix/2019_Bayesian_Melanogaster.
